# Molecular Identification of Bacteria by Total Sequence Screening: Determining the Cause of Death in Ancient Human Subjects

**DOI:** 10.1371/journal.pone.0021733

**Published:** 2011-07-13

**Authors:** Catherine Thèves, Alice Senescau, Stefano Vanin, Christine Keyser, François Xavier Ricaut, Anatoly N. Alekseev, Henri Dabernat, Bertrand Ludes, Richard Fabre, Eric Crubézy

**Affiliations:** 1 Laboratoire AMIS, UMR 5288, Université Toulouse IIII/CNRS/Université de Strasbourg, Toulouse, France; 2 Laboratoire Bio Pole, Toulouse, France; 3 Department of Chemical and Biological Sciences, University of Huddersfield, Huddersfield, United Kingdom; 4 Institut de médecine légale, Strasbourg, France; 5 North-Eastern Federal University, Yakutsk, Sakha (Yakutia) Republic, Russia; 6 Laboratoire de Bactériologie, Faculté de Médecine, Toulouse, France; Monash University, Australia

## Abstract

Research of ancient pathogens in ancient human skeletons has been mainly carried out on the basis of one essential historical or archaeological observation, permitting specific pathogens to be targeted. Detection of ancient human pathogens without such evidence is more difficult, since the quantity and quality of ancient DNA, as well as the environmental bacteria potentially present in the sample, limit the analyses possible. Using human lung tissue and/or teeth samples from burials in eastern Siberia, dating from the end of 17^th^ to the 19^th^ century, we propose a methodology that includes the: 1) amplification of all *16S rDNA* gene sequences present in each sample; 2) identification of all bacterial DNA sequences with a degree of identity ≥95%, according to quality criteria; 3) identification and confirmation of bacterial pathogens by the amplification of the *rpoB* gene; and 4) establishment of authenticity criteria for ancient DNA. This study demonstrates that from teeth samples originating from ancient human subjects, we can realise: 1) the correct identification of bacterial molecular sequence signatures by quality criteria; 2) the separation of environmental and pathogenic bacterial *16S rDNA* sequences; 3) the distribution of bacterial species for each subject and for each burial; and 4) the characterisation of bacteria specific to the permafrost. Moreover, we identified three pathogens in different teeth samples by *16S rDNA* sequence amplification: *Bordetella sp.*, *Streptococcus pneumoniae* and *Shigella dysenteriae*. We tested for the presence of these pathogens by amplifying the *rpoB* gene. For the first time, we confirmed sequences from *Bordetella pertussis* in the lungs of an ancient male Siberian subject, whose grave dated from the end of the 17^th^ century to the early 18^th^ century.

## Introduction

Determination of human bacterial pathogens and their animal vectors is one of the main objectives in paleomicrobiology and in the study of human-environment interactions [1]. Until now, these studies have been mainly carried out on the basis of one essential historical or archaeological observation (texts relating to epidemic diseases, multiple or disaster graves, bone lesions, tissue pathologies) [2]. The well-documented case study is the pandemic plague, researched in sites historically known for the burial of subjects affected by the plague, and where the presence of *Yersinia pestis* has been demonstrated [3], [4], as well as its different clones which spread through Europe between the 15^th^ and 18^th^ centuries [5]. Similarly, human tuberculosis has been confirmed by morphological and molecular methods in an eastern Mediterranean site, dating from 9250–8160 years ago [6]. Recent studies have shown that *Helicobacter pylori* existed in the New World prior to the arrival of Columbus and phylogenetic analysis have indicated that ancient strain clusters are closely related to Asian strains [7], [8]. In these cases historic manuscripts, pathological lesions on bones or specific phylogenetic strains have oriented the choice of pathogen to be researched.

Currently, the genomic approach allows the identification of the different microbial DNA present in biological samples [9]–[11]. However, this analysis not only determines the DNA sequences from sample itself, but also those from the post-burial bacteria. Thus, biological samples contain an assortment of DNA originating from environmental contaminants [12], complicating the identification of human pathogenic bacteria.

**Figure 1:**
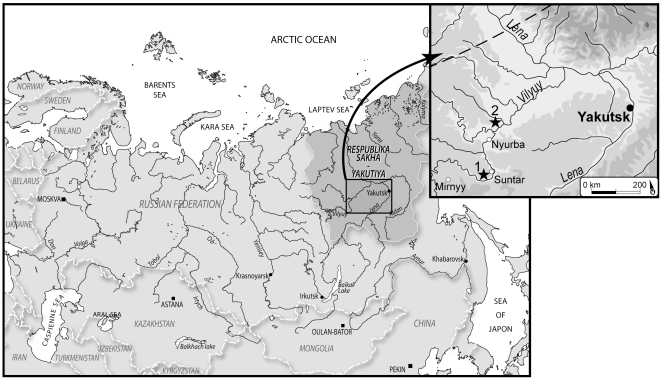
Burial locations in the Vilyuy region, western Yakutia (Eastern Siberia).

The goal of this study was to identify bacterial pathogens in ancient human samples without indications of pathology. For this purpose, we studied five frozen bodies and/or skeletons from Yakutia (eastern Siberia; Fig. 1), dating from between the end of 17^th^ to the 19^th^ centuries, which had been buried in the permafrost. In addition to the good conservation of certain bodies in ice [13], this bacterial research on an autochthonous Yakut population enabled us to analyse the biological consequences of the close interactions between Siberian and Russian (European) populations in a geographically isolated country (see Text S1: contact between autochthonous Siberian and European populations). We applied four methodological objectives to obtain species identifications of human pathogens : 1) the amplification of all *16S rDNA* gene sequences in order to determine the bacterial species present in each sample and to distinguish between environmental and pathogen species; 2) the identification of all bacterial DNA sequences with a degree of identity ≥95%, according to quality criteria, in order to separate different bacterial genus; 3) the identification and confirmation of bacterial pathogens by the amplification of the *rpoB* gene which is more specific to species; and 4) the establishment of authenticity criteria for ancient DNA.

## Results and Discussion

The goal of this study was to identify bacterial pathogens in ancient human samples without indications of pathology. Thus, we analysed ancient tissue samples (teeth and/or lung) from five subjects; two frozen bodies inhumed individually (Fig. S1 and S2) and three skeletons buried in multiple grave (Fig. S3). The four methodological objectives were conducted in a laboratory dedicated to ancient DNA. We used universal primers for the overlapping segments of the bacterial *16S rDNA* gene in order to amplify the environmental and pathogenic bacterial DNA at the same time. First, the amplified sequences were cloned and sequenced, and the quality criteria (described in Materials and Methods 2.2) were applied to identify each sequence using a database. Second, when the bacterial pathogens were detected, we amplified two segments of the *rpoB* gene from each pathogen. If a positive amplified segment was present in the sample, after cloning and sequencing, a phylogenetic test of the sequence was carried out in order to establish the reliability and robustness of the pathogen's identification. Finally, we applied authenticity criteria for ancient DNA which validated the ancient bacterial DNA results.

### 1) Amplification of all *16S rDNA* gene sequences present in each sample

#### 1.1) Human and bacterial DNA amplification

In this first step, we quantified the human nuclear DNA and the total DNA in the ancient samples (teeth and lung tissues) listed in Table 1, in order to evaluate the quality of the human and bacterial DNA available. From the teeth samples of fives subjects, no PCR inhibitors were detected with the co-amplification of nuclear DNA. We noticed that human nuclear DNA was not detected in the OYC sample and its measure of total DNA was the lowest of all the samples.

**Table 1:** Human nuclear DNA quantification by Real Time PCR and Total DNA measures by Nanodrop for each tissue type from each sample.

Samples	Human nuclear DNAMean of quantity of DNA	Total DNAMean of quantity of DNA	Ratiohuman nuclear DNA/total DNA
OYA : teeth	0.023 ng.µl^−1^	8 ng.µl^−1^	0.003
OYB : teeth	0.149 ng.µl^−1^	7.9 ng.µl^−1^	0.019
OYC : teeth	0	0.1 ng.µl^−1^	0
boul.1 : teeth	0.480 ng.µl^−1^	33.15 ng.µl^−1^	0.015
boul.1 : lungs	n.d.	7.9 ng.µl^−1^	n.d.
boul.2 : teeth	0.0139 ng.µl^−1^	n.d.	n.d.
boul.2 : lungs	n.d.	4.9 ng.µl^−1^	n.d
Frequency of PCR inhibitors	0	n.d.	

n.d.: non determined

Human DNA analyses were performed on teeth samples from five subjects for mitochondrial DNA haplogroups, autosomal STR and Y-chromosome STR genotypes. Human DNA profiles confirmed the Siberian origin of these subjects from the Yakut lineage (unpublished data; Keyser and Crubézy [14], [15]).

A bacterial DNA decontamination procedure for the PCR mix was followed [16]; this protocol is highly effective in eliminating background *16S rDNA* contamination [17], whilst preserving the sensitivity of the assay. The PCR blanks and extractions blanks were consistently negative (no amplifications, see Text S1). The amplifications of the four teeth samples were positive for the four overlapping segments P2, M1, M2 and P8 of the *16S rDNA* gene.

### 2) Identification of all bacterial DNA sequences with a degree of identity ≥95%

Several steps were necessary to identify all the *16S rDNA* sequences.

#### 2.1) Determination of the experimental *16S rDNA* sequences according to the defined quality criteria and phylogenetic analysis

A total of 176 clones was obtained and sequenced from the four teeth samples (boul 1, OYA, OYB and OYC). For each tooth sample an average of 38 clones were available with an average of ten clones by *16S rDNA* segment (M2, M1, P8 and P2).

We chose to identify *16S rDNA* sequences in the NCBI refseq_genomic database with a 100% coverage of the query sequence and with a degree of identity ≥95%. On the basis of the quality criteria listed in Table 2 (and referred to as a, b, c1 and c2 in the text), of the total bacterial sequences amplified, 15% did not pass criterion a, and 50–75% did not pass criterion b. On average between 43–62% of the cloned sequences met criteria c1 and c2 (Table 2). Finally, only 67 of the 176 cloned sequences had a degree of identity ≥95% (37%) and only these were used to determine the bacterial sequence composition for each sample. The BLAST research, performed for all the 67 sequences of the *16S rDNA* gene, revealed valid identifications; the various bacteria are listed in Table S1 for all teeth samples (boul 1: S1a; OYA: S1b; OYB: S1c and OYC: S1d). In certain cases, for one experimental sequence we obtained several sequences belonging to different species with equivalent degrees of identity. Therefore, to validate the identification of the obtained experimental sequences we applied the ML method: the JC69 nucleotide substitution model was used in the phylogenetic reconstruction. Several phylogenic trees were constructed but with low resolutions; the DNA region showed a very high degree of identity among the different species and was less informative. Thus, as shown in Table S1 for each tooth sample, we kept several identified sequences with equal degrees of identity.

**Table 2:** Quality criteria applied to the *16S rDNA* sequences from the 176 clones.

	Size of PCR products cloned					
Quality standards	2 strands (F/R)	M2 :93 pb	M1 :146 pb	P8 : 161pb	P2 : 215pb	Total clone number ratio
a: maximum of 0,5% ambiguity from sequences	150	36	31	33	50	150/176
b: sequence homology ≥95%	67	17	16	16	18	67/176
c1: 80%> sequence homology < 95%	61	2	14	17	28	61/176
c2: none identification	22	17	1	0	4	22/176

#### 2.2) Comparison of all the bacterial sequences identified for each subject

**Figure 2:**
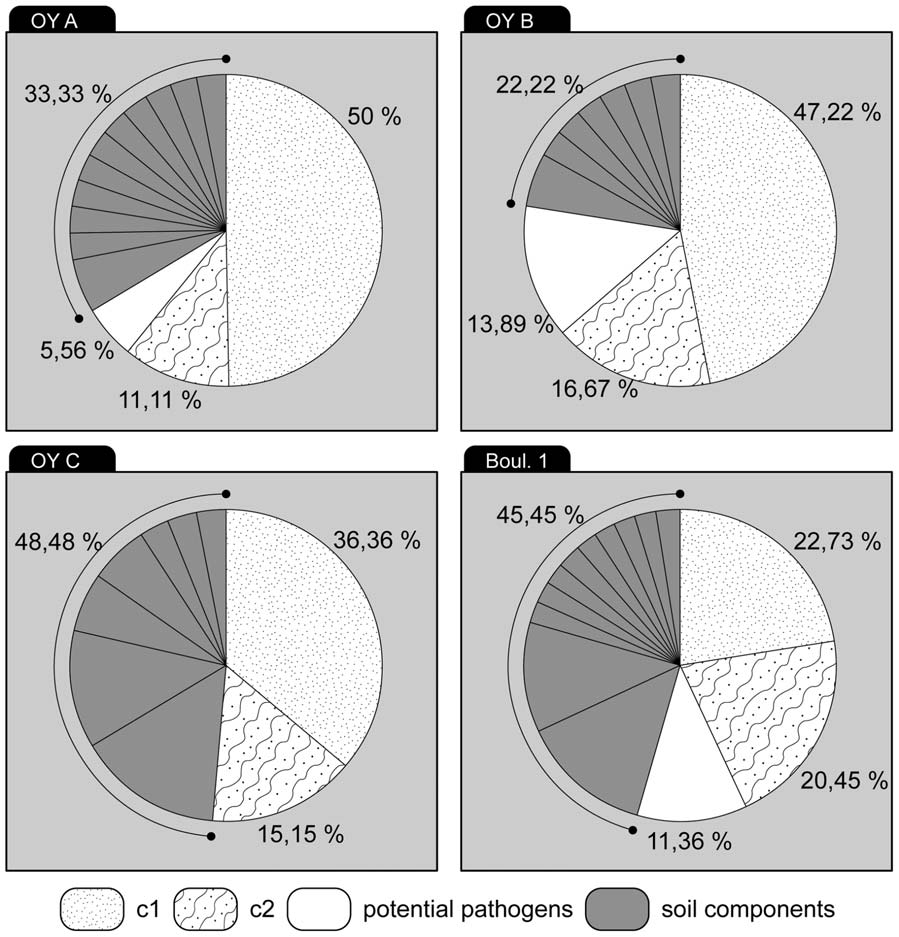
Repartition of environmental, undetermined and potential pathogenic *16S rDNA* sequences in ancient skeletons and frozen bodies.

Figure 2 shows the 176 bacterial *16S rDNA* sequences which constitute the total composition from all tooth samples. The percentage of pathogenic *16S rDNA* sequences identified with a degree of identity ≥95% was 0–14% for the three subjects in Oyogosse Tumula 2 and 11% for the subject boul 1.

For the subject OYC, cavities were present in the teeth. In this context, no bacterial pathogen was identified and a significant number of *Clostridium sp.* sequences originating from the soil (five clones, 31%; Table S1d) were identified. This identification was reinforced by phylogenetic analysis (data not shown). The presence of numerous *Clostridium* bacteria in the OYC tooth sample suggests that bacteria have penetrated and degraded the initial DNA (human and bacteria), explaining why only environmental bacteria have been isolated in this sample.

The comparison of sequence compositions of the four teeth samples is presented in Table S1. *Leifsonia xyli sp.* was identified in the OYA and OYC samples and *Janthinobacterium sp.* or *Herminiimonas arsenicodans sp.* was common to the OYB and OYC subjects. These latter subjects were buried directly in wooden boxes in comparison to the OYA subject who was buried in a coffin, indicating that these bacteria were common in this grave (Table S1b, c and d; Fig.S3). Boul 1 did not present these bacterial sequences; only *Azotobacter vinelandii* or *Xanthobacter autotrophicus* was identified in this sample (Table S1a).

#### 2.3) Environmental bacteria specific to the permafrost

Regarding the identification of *16S rDNA* sequences with a degree of identity greater than 95% (Table S1), the majority of the bacterial pool was environmental bacteria originating from the soil, vegetation or permafrost characteristic of the Arctic and Antarctic [18], [19].

Three samples presented environmental bacterial sequences, including *Pseudomonas sp*, *Xanthomonas sp*, or *Stenotrophomonas sp* (Table S1a, b, and d). These bacteria were targets during the decontaminations of the PCR mixes, and the PCR blank controls always remained negative after amplification, demonstrating their origin from teeth samples (see Text S1). If we consider that all subjects were buried in a wood coffin or box, the presence of these bacterial plant pathogens is not surprising. Moreover, these bacteria are present in the permafrost at the surface or at depths varying from 1.5–3.6 m [19]. For the boul 1 subject, the P8, M1 and P2 overlap sequences confirm the identification with *Pseudomonas sp*. Phylogenetic reconstruction was well-supported and validated the presence of these bacteria (Fig. S4).

We matched one sequence with a close identity to *Mycobacterium marinum* in the OYB subject (Table S1c; [20]). This bacterium has a worldwide geographic distribution and is found in aquatic environments (fresh and saltwater). It is possible that this bacterial sequence is related to the frozen aquatic environment of the permafrost. Another interesting detected bacterium was *Psychrobacter articus* or *Psychrobacter cryohalentis*, which has previously been isolated from the Siberian permafrost [21] and the constructed phylogenetic tree was well-supported (data not shown). Likewise the bacterium *Exiguobacterium sibiricum* was identified in the OYB subject (Table S1c), which has also been isolated from the Siberian permafrost [22]. Two other *16S rDNA* sequences were identified as *Exiguobacterium sibiricum*, but the degrees of identity were 87% in the OYA subject (and are therefore not listed in Table S1b).

### 3) Identification and confirmation of pathogenic bacteria

Three bacterial pathogens were identified based on the *16S rDNA* gene (Table S1): i) in two out of the three subjects from the Oyogosse Tumula grave we identified sequences which had a degree of identity of 97% from *Shigella dysenteriae*: 3 clones from OYA and OYB (Table S1b, c and Text S2c); ii) in one subject (OYB) we revealed one sequence from *Streptococcus pneumoniae* (degree of identity 95%; Tables S1c and Text S2d; another sequence from OYA was found, but with a degree of identity of 92% and is therefore not presented in Table S1b); iii) in the boul 1 subject, we detected five clones of the *16S rDNA* gene sequences which were identified with same degrees of identity of 95–100% for the five species of *Bordetella sp*. and also for *Achromobacter piechaudii* and *Achromobacter xylososidans* (see Table S1a, Text S2a and S2b). For these sequences from boul 1, we noticed that, except for *B. petrii*, other bacteria could be pulmonary pathogens. Because the phylogenetic results did not identify *Achromobacter* versus *Bordetella*, we subsequently sequenced the *rpoB* gene to determine the pathogenic species.

Two specific primer pairs were designed to amplify two different segments of the *rpoB* gene in the three pathogens detected above: *Bordetella sp*., *Streptococcus pneumoniae* and *Shigella dysenteriae* (Table S2).

DNA extracts from teeth of three Oyogosse Tumula subjects did not show any amplifications for *Streptococcus pneumoniae* or *Shigella dysenteriae*; thus these bacteria were not investigated further in this study (see results in Text S3).

Amplifications of the bor1 and bor2 segments were performed to test for *Bordetella sp.* in the lung tissue of the boul 1 sample (Table S2). Amplification of *Bordetella sp.* for segment bor2 was negative (see discussion of result in Text S3).

Amplification of the bor1 segment from boul 1 was performed in two independent PCRs from two independent DNA extracts from lung tissue samples (see Text S1: DNA extracts and multiple independent PCRs). We analysed these PCR products by direct sequencing and subsequently obtained ten clones which were able to be analysed (all sequences and their numbering are presented in Text S4).

In this bor1 segment, one position differed systematically from the reference sequence of *B. pertussis* (NC_002929.2), three positions from *B. bronchiseptica/B. parapertussis*, and six positions differed from *B. petrii* (see Text S4). All the four sequences and ten clones obtained from both lung tissue samples from subject boul 1 were different from the *B. pertussis* strain with regard to one position (C at 2973). The nucleotide C at position 2973 was present in each strand (C on forward and T on reverse) for each clone or independent sequence, and was clearly visible on the electrophoregrams without other peak at this position. This mutation is a transition T→C and can be a post-mortem mutation [24]–[28] or a type 1 damage (T→C/A→G transition), which can represent polymerase errors during the early stages of the PCR process [23]. In contrast, the transition A→G at the position 2965 (type 1 transition) from clone 5 and the insertion of a C between the positions 3022–3023 for clone 6, were never present in other clones or sequences. We took into consideration that PCR amplifications of low copy number templates can generate additional, non-endogenous sequence artefacts, which can easily dominate the products of PCR-amplified ancient DNA [23]–[26]. In our case, we carried out two independent PCRs for both lung extract, and performed sequencing of replicate PCRs and cloning of PCR A1 products to detect single-base errors [27]. All sequences showed a C nucleotide at position 2973, whilst a G nucleotide at position 2965 and a C insertion between the positions 3022–3023 were only shown once. These profiles suggest that the 2973 SNP site in ancient bacteria is C.

**Figure 3:**
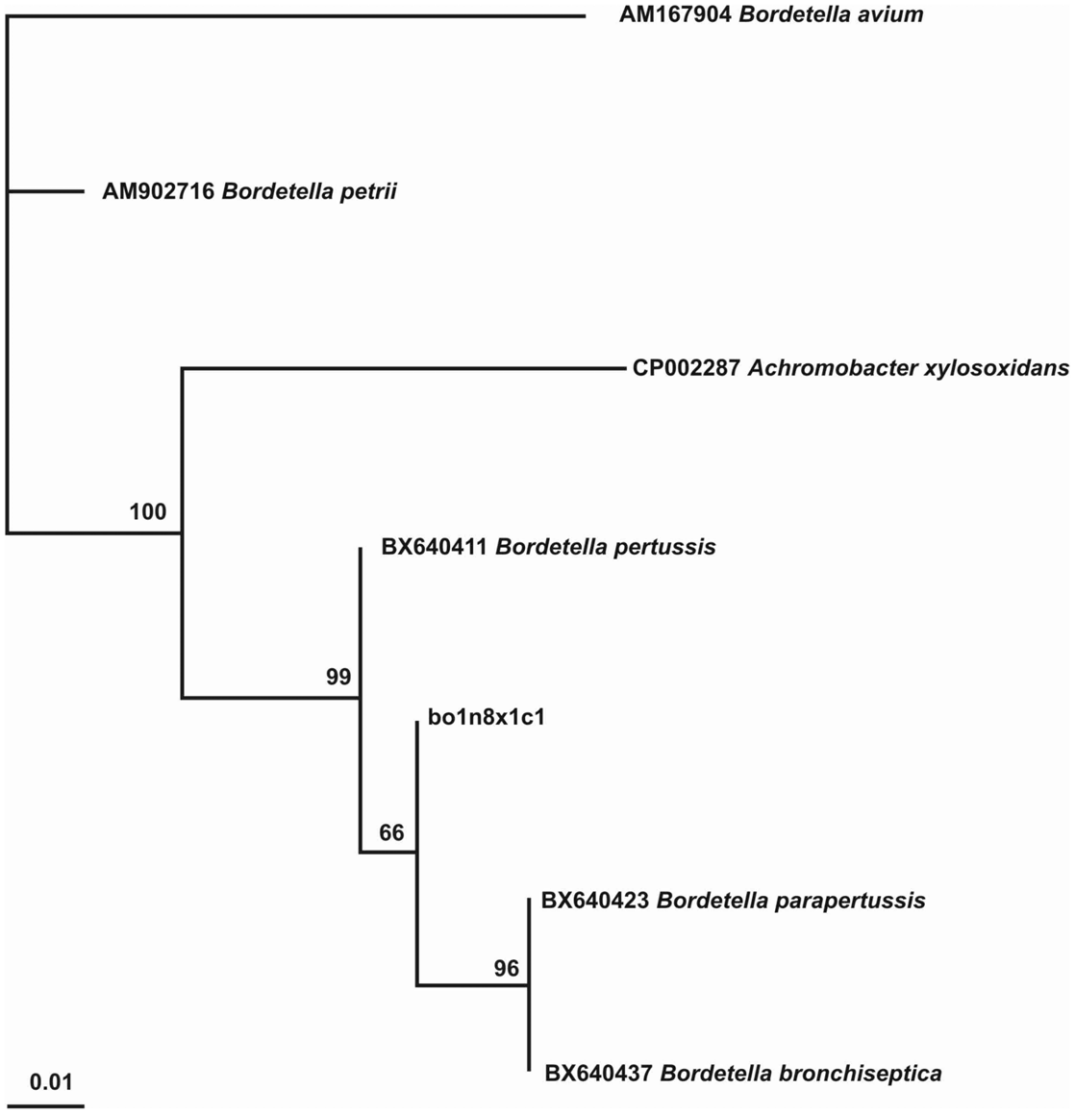
Phylogenetic tree of *rpoB* bor1 segment from boul 1: the phylogeny indicates that the sequence obtained from the ancient boul 1 sample belongs to a species of the genus *Bordetella,* or to particular strains of *B. pertussis*, *B. parapertussis* or *B. bronchiseptica*.

The BLAST research, performed for all the *rpoB* gene clones and sequences, revealed a valid identification with sequences homologous to a species of the *Bordetella* genus. Since all the sequences obtained were identical (eight clones and four independent sequences) we only used one in the phylogenetic reconstruction (bo1n8A1x1c1; Text S4). Using the JC69 nucleotide substitution model, the phylogenetic tree was well-supported, as reported in Figure 3, with BT values ranging from 66–100. The sequence of the *rpoB* bor1 segment from the lung tissue of boul 1 is included in the same branch of *Bordetella pertussis*, *B. parapertussis* and *B. bronchiseptica*, whereas the sequences of *B. petrii* and *B*. *avium* branch are included together at a different position.

To complete the analysis of variation for the bor1 sequences, we searched for conserved domains of the *rpoB* gene in the *B. pertussis* strain (http://www.ncbi.nlm.nih.gov/Structure/cdd/wrpsb.cgi?seqinput=NP_878932.1; [28]–[30]). The bor1 segment is situated between two conserved domains (cd04593 and cd00653; [28]) and only one mutation (at 2973C) differs between our experimental sequences from the subject boul 1 and the reference sequence (*Bordetella pertussis* Tohama I; NC_002929.2).

### 4) Authenticity criteria: controlling for contamination and validation of data

In this study, we considered the criteria for authenticity [31] as essential, but since they cannot always be followed we used the strong logical approach of Gilbert et al.[32] to validate the ancient DNA results. (i) Extensive precautions were taken to avoid contamination by modern DNA, as well as cross-contamination between ancient samples, in the laboratories dedicated to ancient DNA analysis and during PCR cycling (see Text S1 and S3 [33]); (ii) For human and bacterial DNA no amplifications were observed in the extraction blanks or PCR blanks, demonstrating no contamination during pre-PCR preparation and analytical steps. Likewise, when bacterial DNA was studied, additional precautions were taken, according to the specific tissue (lungs and teeth) and the issues of modern bacteria (see text S3 [33]); (iii) Reproducibility of *rpoB* bor1 segments with multiple independent PCRs and cloning of PCR products were demonstrated (Text S4); (iv) Biochemical preservation was evaluated; histological sections were performed on the different tissues of the frozen bodies (boul 1 and boul 2) and were consistent with conservation in permafrost; sequences of poor quality or with lower degrees of identity with database sequences were excluded from analyses (see Table 2); (v) Quantitative PCR performed on human DNA was reliable according to the expected values for ancient human DNA (Table 1 and [34]) and showed an absence of inhibitor; (vi) Human and bacterial DNA analyses of samples taken from the frozen bodies were performed in independent ancient DNA laboratories, and were determined by reproducible PCR results performed on both strands of the DNA from multiple extractions (Texts S1 and S4; [14]). The allelic human profiles (mitochondrial DNA, autosomal STR and Y-chromosome STR) obtained were not mixtures of different individuals' DNA and no profiles matched those of the researchers involved in the handling of the bones or DNA samples. Moreover, the bacterial DNA identified, including *Exiguobacterium sibiricum*, *Psychrobacter articus* or *Psychrobacter cryohalentis*, were characteristic of the ecological environment of the human burials in a permafrost environment and reinforce the origin of the bacterial DNA sequences.

In addition to the criteria selected by [32], we have applied additional criteria of authenticity to validate the reliability of our results: (a) The genetic characteristics of the Yakuts are well-established [14] and human DNA results confirmed the Siberian origin of subjects (Unpublished data; Keyser and Crubézy); (b) Identification of the same *16S rDNA* sequences in the teeth samples of three subjects of Oyogosse Tumula: environmental bacteria (*Leifsonia xyli sp.* and *Janthinobacterium sp.* or *Herminiimonas arsenicodans sp.*), and particularly the pathogenic bacteria (*S. dysenteriae* and *S. pneumoniae*) detected in OYA and OYB (see also Text S3), and its absence in the boul 1 sample. Similarly, these differences were found in the pathogenic sequences identified in the teeth sample from the Boulgouniak 1 subject and were not present in the three subjects from Oyogosse Tumula. The differential detection of pathogens demonstrated that no cross-contamination between samples occurred during excavations or pre-PCR preparations; (c) The subject OYC presented cavities in most of the teeth samples, but human DNA was not found. Likewise, no bacterial pathogens were found in this sample, demonstrating no cross-contamination with other samples; (d) We have only considered bacterial sequences that matched to the sequences recorded in the NCBI refseq_genomic database with a degree of identity ≥95%, and only phylogenetic tree reconstructions with robust BT values were considered as informative. The consistency of results may be validated by sequences from *Bordetella* genus. Indeed, only boul 1 presented sequences from the *Bordetella* genus in the teeth (via the *16S rDNA* gene; Table S1a; Text S2a and S2b) with degree of identity of 95–100%; sequences were absent in other teeth samples without the use of positive control in this step. We tested the lung tissue samples from boul 1 and boul 2 and the sample boul 2 never amplified *rpoB* segments.

### Conclusion

In this study we demonstrate that bacterial pathogens can be identified in ancient human subjects without preliminary observations such as historical texts or pathological lesions on bodies. Our methodology defined in four steps allowed us to identify sequences of pathogenic bacteria, principally from *Bordetella pertussis.* Indeed, the *rpoB* segment, as well as the two P2 and three M1 *16S rDNA* segments, appear to be sequences of a *B. pertussis* strain present in a male subject of the Siberian elite class dating from the end of the 17^th^ century to the early 18^th^ century. The small ancient *B. pertussis* sequences that were found are very similar to the modern-day *B. pertussis* strain (95–100% of degree of identity).

*B. pertussis* (whooping-cough) is a highly contagious disease in human populations that can cause death, either directly or through its secondary infections [35]. Humans are the only host of the bacteria, because all *Bordetella* species (except the environmental strain, *B. petrii* [36]) have limited viability in environments outside their host, since they are typically sensitive to UV light, extreme temperatures and pH. In populations with the disease children are affected the most, and if they survive to adulthood they can be carriers and transmit the bacteria to non-immune subjects.

In Yakutia, we can assume that contact between European (notably merchants) and autochthonous populations was at the origin of a series of epidemics. However, *B. pertussis* has not been reported in texts despite its presence in a Yakut adult 100 years after the European arrival. To confirm this first result, future research will explore additional graves of the Siberian elite from this period, to evaluate the impact of pathogens on ancient Yakut populations.

## Materials and Methods

Three laboratories were involved in this work. Two laboratories (lab.1 and lab.3) have a dedicated, physically-isolated laboratory for ancient DNA; lab.1 carried out independent DNA extractions from lung and teeth samples and human DNA analysis and lab.3 performed pre-PCR manipulations on ancient bacterial DNA. One modern DNA laboratory: lab.2 performed and tested the molecular set-up for each step of our methodology, notably preliminary decontamination of PCR products. Lab.3 has a modern DNA laboratory for post-PCR handling, which is physically separated in another building from the laboratory dedicated to ancient DNA.

### 1) Amplification of all *16S rDNA* gene sequences present in each sample

#### 1.1) Samples

Subjects were buried in the Vilyuy region (western Yakutia, eastern Siberia). The first two graves were found in the permafrost, and each contained one naturally mummified subject. In the first grave, (boul 1: Fig.S1; star 1, Fig. 1), the subject was male with characteristic Asian features. The second grave (boul 2: Fig.S2; star 1, Fig. 1) containing a female subject was situated fifteen metres away from the first grave. Both these subjects possessed artefacts signifying wealth and membership to the Siberian elite class. In contrast, the third grave (star 2, Fig. 1) was a multiple burial of three anatomically European subjects in skeletal states buried in ice (named OYA, OYB and OYC respectively; Fig.S3).

For these five subjects, no anatomic or pathologic lesions were found. Samples from teeth and/or lung tissues were taken from the subjects *in situ*. Genetic analysis of human DNA was possible for the teeth samples from all the subjects, while analysis of the *16S rDNA* bacterial gene was only performed on the teeth of four subjects (boul 1 and OYA, OYB, OYC). Research on the *rpob* bacterial gene for the *Bordetella* genus was only performed on the lung tissue samples from boul 1 and boul 2 subjects, due to tissue conservation in the permafrost.

#### 1.2) Sample preparation and DNA extraction

All preparation and extraction procedures were conducted in lab. 1 according to teeth protocols published previously [37]. Aliquots of 0.3 g of powder from the totality of the teeth samples were used to obtain DNA extracts for each subject. For the lung tissue samples aliquots of 0.6 g was used for each extraction (boul 1 and boul 2), following the protocol described in [38]. Two independent extractions and blank extractions were performed for each subject's sample (teeth or lung tissue; see Text S1 for further explanations of the control and decontamination procedures).

#### 1.3) Total DNA Nanodrop measures and human nuclear DNA quantification

In lab.1, human nuclear DNA quantification was carried out on the teeth samples of the five subjects using the Quantifiler® Human DNA Quantification Kit (Applied Biosystems) described in [14]. In addition to the quantification of nuclear DNA, the presence of PCR inhibitors was determined with the co-amplification of an internal PCR control included in each reaction. The total DNA in each sample was measured by Nanodrop (Labtech; lab.3).

#### 1.4) Analysis of ancient human DNA

The AmpFlSTR® Y-Filer™ kit (17 loci) was used for Y-chromosome STR amplification and two overlapping fragments of the HVS-1 region were amplified, as described in [14]. Autosomal STRs were amplified using the AmpFlSTR® Profiler Plus™ Kit (Applied Biosystems) as performed in [34].

#### 1.5) Bacterial composition of each sample by *16S rDNA* gene amplification

PCR procedures on ancient bacterial DNA were performed in lab.3 dedicated to ancient DNA. The four primer pairs for the *16S rDNA* gene are listed in Table S2. These overlapping primer couples were chosen because they each permit a segment amplification of 90 at 215 bp, and together they analyse a 477 bp segment of the *16S rDNA* gene including the variable regions V6, V7 and V8 and a part of the conserved regions C5, C6 and C7 [11].

The PCR mix was prepared as follows for a final volume of 20 µl: 1X of buffer, 0.5 µl of Taq LD, 2.5 mM of MgCl2, 200 µM of dNTPs (Applied Biosystem), 800 nM of each primer (Invitrogen) and 1X of Buffer 4 (Biolabs). One decontamination step was also systematically made, following [16]. After these preparation steps the PCR mix was divided into each PCR tube under the laminar flow hood 1. Subsequently, 2 µl of DNA extract was added to each PCR tube under the laminar flow hood 2 (see Text S1). PCRs were performed under the following conditions: (i) initial denaturation step at 95°C for 10 min; (ii) 40 cycles of denaturation at 95°C for 30 secs; (iii) hybridization at 60°C (for all primer couples) for 45 secs; (iv) extension at 72°C for 90 secs; and (v) final extension at 72 °C for 1 min in a Biometra thermocycleur (Archamps). All PCR products were cloned and sequenced as described in Text S1.

### 2) Identification of all bacterial DNA sequences with a degree of identity ≥95%

#### 2.1) Sequences and databases

Electrophoregrams were analysed with Sequence Scanner v1.0 software (Applied Biosystems). Consensus sequences, obtained from the comparison of both strands for each sequence, were compared using BLASTN on the whole NCBI refseq_genomic database (http://www.ncbi.nlm.nih.gov/blast/Blast.cgi) and on the DNA Data Bank of Japan (http://blast.ddbj.nig.ac.jp/).

#### 2.2) Quality criteria

Quality criteria were followed during the examination of the *16S rDNA* sequences. These criteria were applied to the experimental *16S rDNA* and *rpoB* sequences which matched the database sequences in order to validate their degree of identification. These criteria are different to the authenticity criteria used to certify ancient DNA sequences. More precisely, the criteria: (a) correspond to a maximum sequence ambiguity of 0.5% during the reading of the electropherograms; (b) correspond to a 100% coverage and a degree of identity ≥95% of the experimental sequence matched to the bacterial sequences recorded in the database, estimated by BLASTN on the complete NCBI refseq_genomic database and; (c) were divided into two groups: a low sequence identity of 80–95% between the experimental sequence and the database sequences (c1), and no identification of the experimental sequence in the current database (c2).

The NCBI Conserved Domain Structure database was used to determine the conserved domains of the *Bordetella pertussis rpoB* gene. (http://www.ncbi.nlm.nih.gov/cdd; [28]–[30]).

#### 2.3) Phylogenetic analyses

Sequences were automatically extracted from the available databases using BLAST searches [39]. Multiple alignments were done using CLUSTALW [40]. The final alignment was refined manually and used in the subsequent analyses. A preliminary quartet puzzling analysis was performed with the Treepuzzle program [41], [42] to test whether a phylogenetic approach could be applied to the original data set. Phylogenetic studies were performed according to the maximum likelihood method (ML) with the PHYML 2.4 program [43]. The JC69 nucleotide substitution model [44] was used in the reconstruction. Nonparametric bootstrap resampling (BT) [45] was performed with 1000 replicas to test the robustness of the tree topology. The phylogenetic tree was visualized with the Fig Tree 1.1.1 program (http://tree.bio.ed.ac.uk/software/figtree/).

### 3) Identification and confirmation of pathogen bacteria by *rpoB* gene amplification

All primers for the three species of pathogenic bacteria are listed in Table S2. These primers are specific to the *rpoB* gene of each pathogen, and thus should not hybridize aspecifically to the environmental bacteria overrepresented in quality and quantity (see Text S1 for the use of extraction blanks, PCR blanks). PCR mixes were performed in a final volume of 25 µL: 1X of buffer, 2.5 mM of MgCl2, 200 µM of dNTPs, 2.5 U of Taq Gold (Applied Biosystems) and 400 mM of each primer. In the final step, 2.2 µL of the ancient DNA sample was added under the laminar flow hood 2. PCRs were performed under the following conditions: (i) initial denaturation step at 95°C for 5 min; (ii) 40 cycles of denaturation at 95°C for 30 secs; (iii) hybridization for 20 secs at 65°C for bor1, at 68°C for bor2 *Bordetella* primer couples, at 64°C for both St1 and St2 *Streptococcus pneumoniae* primer couples, at 67°C for Sg1 and at 67°C for Sg2 *Shigella dysenteriae* primer couples; (iv) elongation step at 72°C for 15 secs; and (v) a final extension at 72°C for 4 min.

Independent PCR amplifications of both segments of the *B. pertussis rpoB* gene were performed on two extractions from the teeth and lung tissue samples of boul 1. When PCR amplification was positive, PCR products were directly i) sequenced and/or ii) cloned and sequenced as described in Text S1.

### 4) Authenticity criteria: controlling for contaminations and validation of data

The following steps were taken in the study: i) Considering the possibility of modern DNA contamination during excavations [46], samples were collected with extensive precautions as described in [14]; (ii) The preparation of samples and DNA extractions were conducted in a sterile room dedicated solely to ancient DNA work (lab.1). Similarly, steps prior to bacterial DNA amplifications were performed in a pre-PCR area of a laboratory dedicated to ancient DNA work (lab.3), physically separated from the modern DNA work. Decontamination and work conditions in the ancient DNA laboratories are described in Text S1. Amplification, cloning and sequencing were carried out in the post-PCR lab.3; (iii) All steps were monitored by blank extractions and blank PCR controls; (iv) Samples were cloned and sequenced to detect heterogeneous sequences due to DNA degradation or contamination; (v) We checked the results of different samples to evaluate no contamination of human or bacterial DNA between the subjects; (vii) Independent PCR replications were carried out for human and bacterial DNA analyses (lab.1 and lab.3) and reproducible PCR results were performed on both strands of the DNA from multiple extractions, firstly in teeth samples, and when tissues were available, in lung samples; (viii) Quantitative human DNA real-time PCR was carried out on samples to ensure appropriate levels of DNA quantity and quality and to assess the presence of PCR inhibitors; and (ix) Preservation of tissues was evaluated through histological sections.

## Supporting Information

Figure S1 Boulgouniak 1 grave. The body of the Siberian man was partially preserved by the permafrost.
(TIF)

Figure S2 Boulgouniak 2 grave. The body of a Siberian woman was in the ice.
(TIF)

Figure S3 Oyogosse Tumula 2 multiple grave. Ice was present in the bottom of coffin.
(TIF)

Figure S4 Reconstruction of overlapping segments from *Pseudomonas sp*. In particular the phylogenetic relationships of the boul 1 subject sequences, with overlapping P8, M1 and P2 segments, indicate that these sequences belong to a *Pseudomonas* species close to *P. aeruginosa*. Bo6-P8: clone 6 from boul 1 sample for P8 segment; bo1-M1: clone 1 from boul1 sample from M1 segment; bo15-P2: clone 15 from boul 1 sample from P2 segment.
(TIF)

Table S1 Composition of the soil component and potential pathogen sequences with degrees of identities ≥95%, for each subject.
(DOC)

Table S2 PCR primer sequences used in this study.
(DOC)

Text S1 Ancient DNA analysis informations
(DOC)

Text S2 Alignments of the *16S rDNA* sequences from boul 1, OYA and OYB subjects and the matched sequences recorded in the Genbank database.
(DOC)

Text S3 Alignments of the *rpoB* sequences from boul 1 subject with the sequences of *B. pertussis*, *B. bronchiseptica*, *B. parapertussis*, and *B. petrii* recorded in the Genbank database.
(DOC)

Text S4 Supplementary data of the obtained results from *B. pertussis*, *S. dysenteriae* and *S. pneumoniae*.
(DOC)
