# Supplementary figures and images for: Molecular Identification of Bacteria by Total Sequence Screening: Determining the Cause of Death in Ancient Human Subjects
Source: PLoS One. 2011 Jul 13;6(7):e21733. doi: 10.1371/journal.pone.0021733 (PMC3135582; doi:10.1371/journal.pone.0021733)

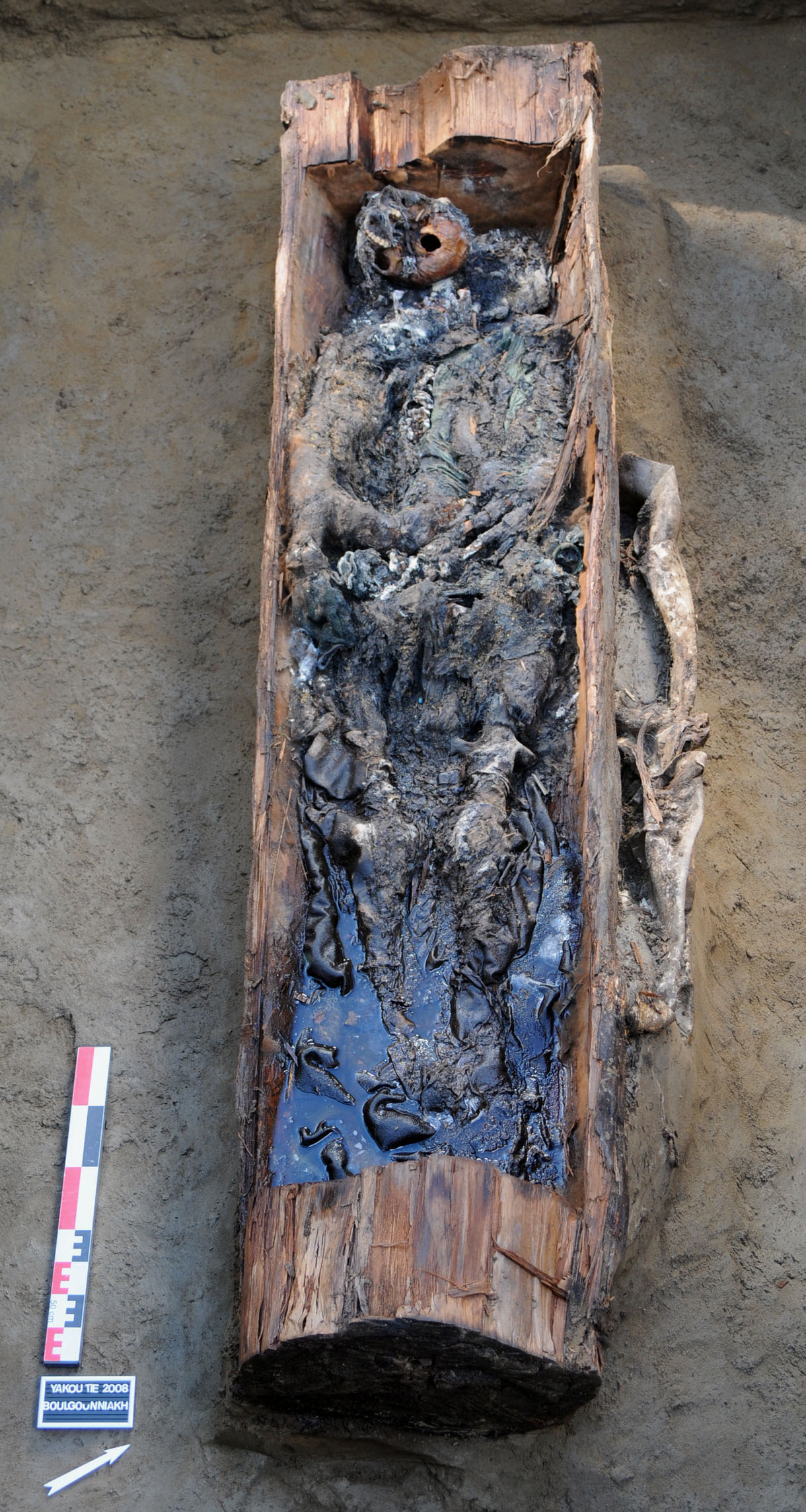

Supplement: Figure S1 — Boulgouniak 1 grave. The body of the Siberian man was partially preserved by the permafrost. (TIF) [file pone.0021733.s001.tif]

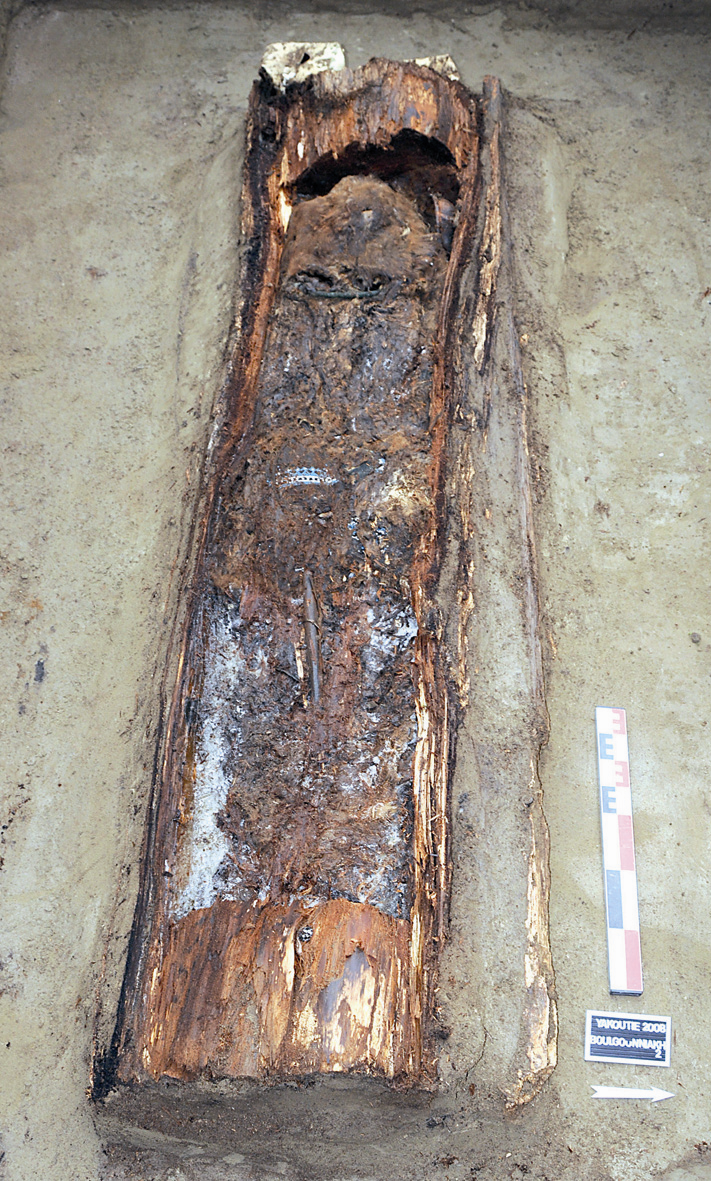

Supplement: Figure S2 — Boulgouniak 2 grave. The body of a Siberian woman was in the ice. (TIF) [file pone.0021733.s002.tif]

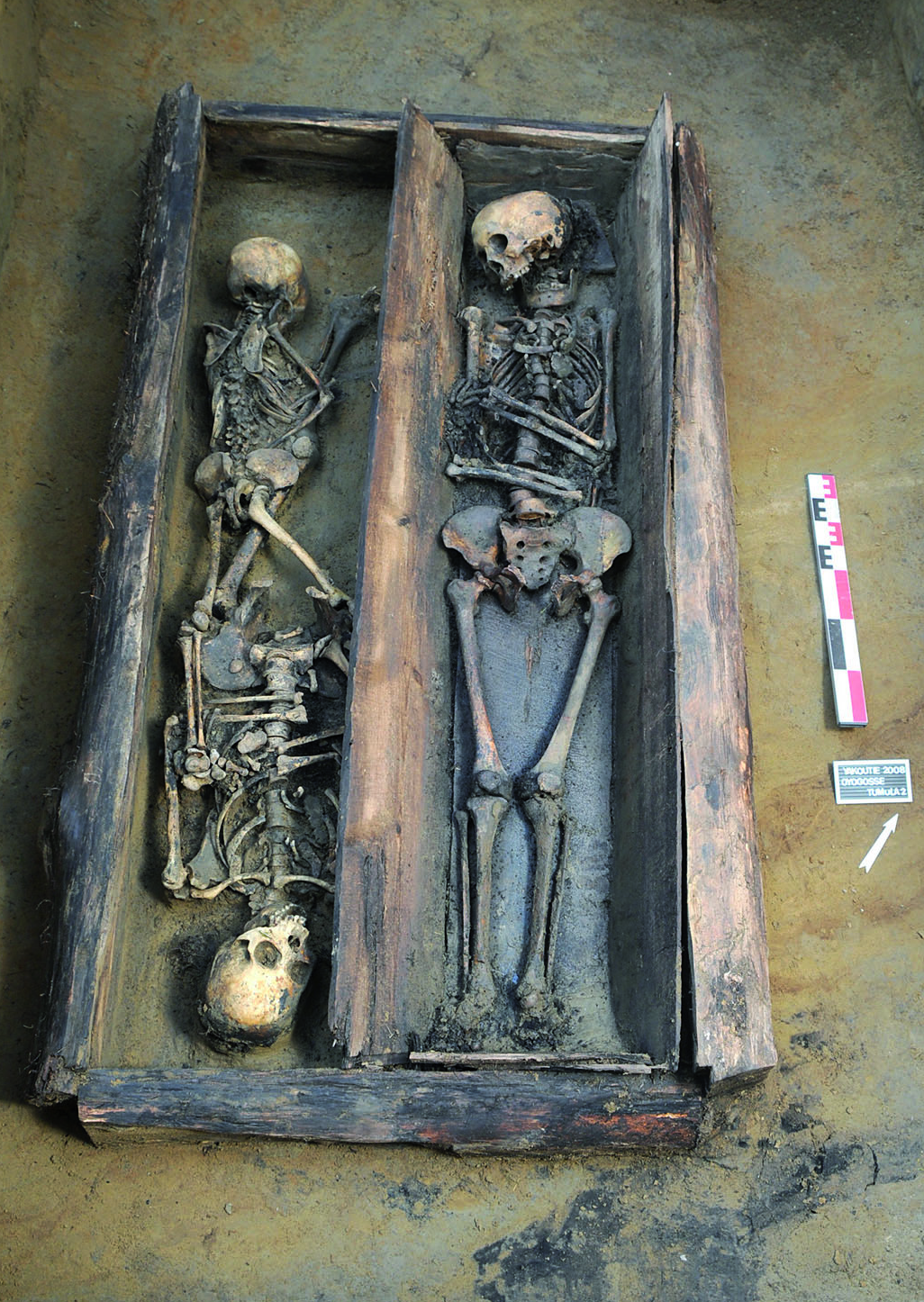

Supplement: Figure S3 — Oyogosse Tumula 2 multiple grave. Ice was present in the bottom of coffin. (TIF) [file pone.0021733.s003.tif]

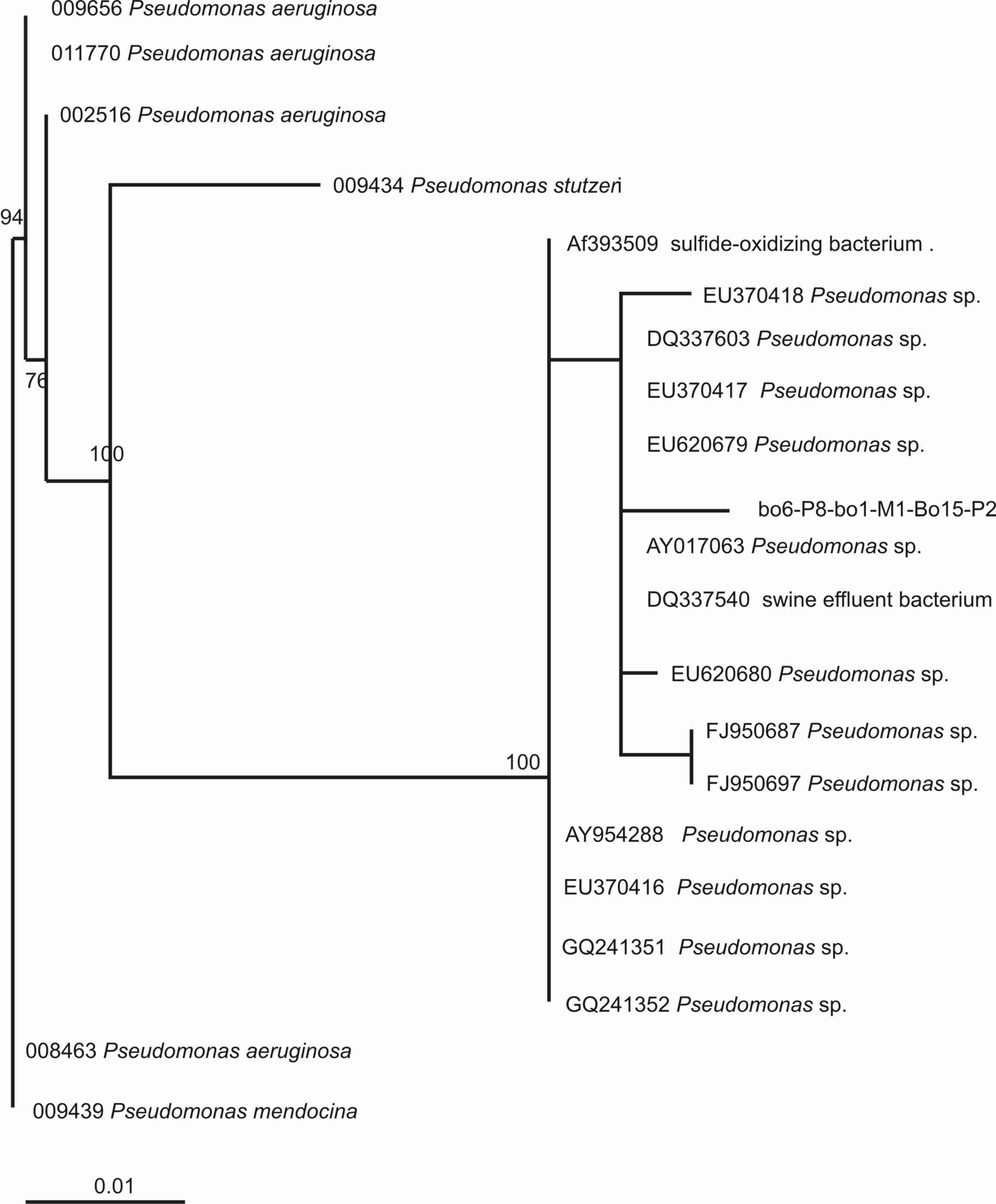

Supplement: Figure S4 — Reconstruction of overlapping segments from Pseudomonas sp. In particular the phylogenetic relationships of the boul 1 subject sequences, with overlapping P8, M1 and P2 segments, indicate that these sequences belong to a Pseudomonas species close to P. aeruginosa. Bo6-P8: clone 6 from boul 1 sample for P8 segment; bo1-M1: clone 1 from boul1 sample from M1 segment; bo15-P2: clone 15 from boul 1 sample from P2 segment. (TIF) [file pone.0021733.s004.tif]
