# Supplementary material for: Molecular Identification of Bacteria by Total Sequence Screening: Determining the Cause of Death in Ancient Human Subjects
Source: PLoS One. 2011 Jul 13;6(7):e21733. doi: 10.1371/journal.pone.0021733 (PMC3135582; doi:10.1371/journal.pone.0021733)
Supplement: Table S1 — Composition of the soil component and potential pathogen sequences with degrees of identities ≥95%, for each subject. (DOC) [file pone.0021733.s005.doc]

**Table S1a: Composition of the soil component and potential pathogen sequences with degrees of identities ≥ 95%, for the tooth sample of boul 1 subject.**

| **16S rDNA segments** | **Sequences identity on NCBI refseq_genomic database** | **Number of clones** | **Maximum identity (%)** |
| --- | --- | --- | --- |
| P2 | NC_014924.1 *Pseudoxanthomonas suwonensis* | 2 | 95 |
|  | NC_002678.2 *Mesorhizobium loti* | 1 | 99 |
|  | NC_012560.1 *Azotobacter vinelandii* | 2 | 95 |
|  | NC_009720.1 *Xanthobacter autotrophicus* | 1 | 96 |
|  | *Achromobacter* or *Bordetella* * | 2 | 95-96 |
|  |  |  |  |
| M1 | *Pseudomonas* § | 1 | 99 |
|  | NC_011027.1 *Chlorobaculum parvum* | 2 | 95 |
|  | *Achromobacter* or *Bordetella* # | 3 | 99-100 |
|  |  |  |  |
| P8 | NC_011770.1 *Pseudomonas* | 6 | 97 |
|  | NC_009848.1 *Bacillus pumilus* | 1 | 98 |
|  |  |  |  |
| M2 | *Xanthomonas* or *Stenotrophomonas* or *Xylella* ‡ | 5 | 97 |

* including 100% of query coverage and maximum degree of identity of different species: NZ_GG770424.1 *Achromobacter piechaudii*; NC_014640.1 *Achromobacter xylosoxidans*; NC_010645.1 *Bordetella avium*; NC_002927.3 *Bordetella bronchiseptica*; NC_002928.3 *Bordetella parapertussis*; NC_010170.1 *Bordetella petrii*; NC_002929.2 *Bordetella pertussis*.

§ including 100% of query coverage and maximum degree of identity of different species:

NZ_GG774664.1 *Pseudomonas savastanoi*; NC_007492.2 *Pseudomonas fluorescens*; NC_002947.3 *Pseudomonas putida*.

# including 100% of query coverage and maximum degree of identity of different species:

NZ_GG770424.1 *Achromobacter piechaudii*; NC_014640.1 *Achromobacter xylosoxidans*; NC_010645.1 *Bordetella avium*; NC_002927.3 *Bordetella bronchiseptica*; NC_002928.3 *Bordetella parapertussis*; NC_010170.1 *Bordetella petrii*; NC_002929.2 *Bordetella pertussis*.

‡ including 100% of query coverage and maximum degree of identity of different species:

NC_003902.1 and NC_007086.1 and NC_007508.1 and NC_010688.1 and NZ_GG699328.1 and NZ_GG699422.1 *Xanthomonas campestris*; NC_013722.1 *Xanthomonas albilineans*; NC_006834.1 and NC_007705.1 and NC_010717.1 *Xanthomonas oryzae*; NC_003919.1 *Xanthomonas axonopodis*; NC_010943.1 and NC_011071.1 *Stenotrophomonas maltophilia*; NC_004556.1 and NC_010513.1 and NC_010577.1 *Xylella fastidiosa*.

**Table S1b: Composition of the soil component and potential pathogen sequences with degrees of identities ≥ 95%, for the tooth sample of OYA subject.**

| **16S rDNA segments** | **Sequences identity on NCBI refseq_genomic database** | **Number of clones** | **Maximum identity (%)** |
| --- | --- | --- | --- |
| P2 | NC_008825.1 *Methylibium petroleiphilum* or NC_010524.1 *Leptothrix cholodnii* | 1 | 98 |
|  | NC_006087.1 *Leifsonia xyli* | 1 | 98 |
|  | NC_014924.1 *Pseudoxanthomonas suwonensis* or NC_010943.1 *Stenotrophomonas maltophilia* | 1 | 95 |
|  | NC_014659.1 *Rhodococcus equi* or NC_012522.1 *Rhodococcus opacus* | 1 | 99 |
|  |  |  |  |
| M1 | *Pseudomonas* ‡ | 1 | 99 |
|  |  |  |  |
| P8 | NZ_DS989898.1 *Brevundimonas sp*. or NC_014100.1 *Caulobacter segnis* | 1 | 98 |
|  | NZ_CM001020.1 *Pseudomonas aeruginosa* or NC_009439.1 *Pseudomonas mendocina* | 1 | 97 |
|  | NZ_GL455582.1 *Enterococcus faecalis* or NC_007576.1 *Lactobacillus sakei* | 1 | 99 |
|  |  |  |  |
| M2 | NC_007606.1 *Shigella dysenteriae* | 2 | 97 |
|  | NC_007517.1 *Geobacter metallireducens* or NC_002939.4 *Geobacter sulfurreducens* | 1 | 97 |
|  | NC_008344.1 *Nitrosomonas eutropha* | 1 | 97 |

‡ including 100% of query coverage and maximum degree of identity of different species: NZ_GG774664.1 *Pseudomonas savastanoi*; NC_007492.2 *Pseudomonas fluorescens*; NC_002947.3 *Pseudomonas putida*.

**Table S1c: Composition of the soil component and potential pathogen sequences with degrees of identities ≥ 95%, for the tooth sample of OYB subject.**

| **16S rDNA segments** | **Sequences identity on NCBI refseq_genomic database** | **Number of clones** | **Maximum identity (%)** |
| --- | --- | --- | --- |
| P2 | NC_010612.1 *Mycobacterium marinum* | 1 | 95 |
|  | NC_009659.1 *Janthinobacterium sp. Marseille* or NC_009138.1 *Herminiimonas arsenicoxydans* | 1 | 98 |
|  |  |  |  |
| M1 | NZ_CH672415.1 *marine actinobacterium* | 1 | 100 |
|  | NC_007969.1 *Psychrobacter cryohalolentis* or NC_007204.1 *Psychrobacter arcticus* | 2 | 100 |
|  | NC_010556.1 *Exiguobacterium sibiricum* | 1 | 95 |
|  | NC_014923.1 *Mesorhizobium ciceri* | 3 | 100 |
|  |  |  |  |
| P8 | *Streptococcus pneumoniae* * | 1 | 96 |
|  | *Parvibaculum* or *Mesorhizobium* § | 1 | 97 |
|  |  |  |  |
| M2 | NC_007404.1 *Thiobacillus denitrificans* | 1 | 98 |
|  | NC_007606.1 *Shigella dysenteriae* | 1 | 97 |

***** including 100% of query coverage and maximum degree of identity of different species: NC_011072.1 and NC_003028.3 and NC_010582.1 *Streptococcus pneumoniae*; NC_010380.1 *Streptococcus pneumoniae Hungary*.

§ including 100% of query coverage and maximum degree of identity of different species: NC_014923.1 *Mesorhizobium ciceri*; NC_009719.1 *Parvibaculum lavamentivorans*; NC_002678.2 *Mesorhizobium loti*.

**Table S1d: Composition of the soil component and potential pathogen sequences with degrees of identities ≥ 95%, for the tooth sample of OYC subject.**

| **16S rDNA segments** | **Sequences identity on NCBI refseq_genomic database** | **Number of clones** | **Maximum identity (%)** |
| --- | --- | --- | --- |
| P2 | NC_009659.1 *Janthinobacterium sp. Marseille* or NC_009138.1 *Herminiimonas arsenicoxydans* | 2 | 100-99 |
|  | *Xanthomonas* * | 1 | 96 |
|  | NC_013530.1 *Xylanimonas cellulosilytica* or NC_006087.1 *Leifsonia xyli* | 1 | 98 |
|  |  |  |  |
| M1 | NC_009617.1 *Clostridium beijerinckii* | 1 | 98 |
|  | *Methylobacterium* § | 1 | 97 |
|  |  |  |  |
| P8 | *Clostridium* ¶ | 4 | 97-96 |
|  |  |  |  |
| M2 | *Xanthomonas* ou *Stenotrophomonas* # | 2 | 97 |
|  | *Acinetobacter sp.* ‡ | 4 |  |

***** including 100% of query coverage and maximum degree of identity of different species: NZ_GG699422.1 and NZ_GG699328.1 *Xanthomonas campestris*; NC_010717.1 and NC_007705.1 *Xanthomonas oryzae*; NC_007508.1 *Xanthomonas campestris*; NC_006834.1 *Xanthomonas oryzae*; NC_003919.1 *Xanthomonas axonopodis*.

§ including 100% of query coverage and maximum degree of identity of different species: NC_012988.1 and NC_012808.1 *Methylobacterium extorquens*; NC_011757.1 *Methylobacterium chloromethanicum*; NC_010725.1 *Methylobacterium populi*; NC_010510.1 and NC_010505.1 *Methylobacterium radiotolerans*; NC_010172.1 *Methylobacterium extorquens*.

**¶** including 100% of query coverage and maximum degree of identity of different species: NZ_GG770705.1 and NZ_GG770681.1 *Clostridium carboxidivorans*; NC_009617.1 *Clostridium beijerinckii*.

**#** including 100% of query coverage and maximum degree of identity of different species: NZ_GG699422.1 and NZ_GG699328.1 *Xanthomonas campestris*; NC_013722.1 *Xanthomonas albilineans*; NC_011071.1 and NC_010943.1 *Stenotrophomonas maltophilia*; NC_010717.1 *Xanthomonas oryzae*;

‡ including 100% of query coverage and maximum degree of identity of different species: NZ_GG704113.1 and NZ_GG703914.1 and NZ_GG704236.1 *Acinetobacter sp*.
