# Supplementary material for: Molecular Identification of Bacteria by Total Sequence Screening: Determining the Cause of Death in Ancient Human Subjects
Source: PLoS One. 2011 Jul 13;6(7):e21733. doi: 10.1371/journal.pone.0021733 (PMC3135582; doi:10.1371/journal.pone.0021733)
Supplement: Table S2 — PCR primer sequences used in this study. (DOC) [file pone.0021733.s006.doc]

**Table S2: PCR primer sequences used in this study.**

| 16S rRNA bacterial gene from Horz et al. (2005) | primers | sequences | *E. Coli* positions | segment name |
| --- | --- | --- | --- | --- |
|  | M1-fw | GTGSTGCAYGGYTGTCGTCA | 1048-1067 | M1 |
|  | M1-rev | ACGTCRTCCMCCCTTCCTC | 1175-1194 |  |
|  | P2-fw | GAGGAAGGHGHGGAHGACGT | 1175-1194 | P2 |
|  | P2-rev | AGHCCCGHGAACGTATTCAC | 1371-1390 |  |
|  | P8-fw | TGGAGCATGTGGTTTAATTCGA | 943-964 | P8 |
|  | P8-rev | TGCGGGACTTA CCCAACA | 1083-1101 |  |
|  | M2-fw | CCATGAAGTCGGAATCGCTAG | 1327-1347 | M2 |
|  | M2-rev | ACTCCCATGGTGTGACGG | 1403-1420 |  |
| this study  rpoB bacterial gene for *Bordetella pertussis*. |  |  | *Bordetella pertussis* Tohama I positions |  |
|  | B1F | CGTTATCGCCAGGACCTCAA | 2880-2900 | bor1 |
|  | B1R | GCCAGGTAGGCCTTGGTCAA | 3025-3006 |  |
|  | B2F | GGCCCGTACTCGCTGGTTA | 3828-3846 | bor2 |
|  | B2R | GTGATGTCGTCGGACTTCA | 3979-3966 |  |
| rpoB bacterial gene for *Shigella dysenteriae* |  |  | *Shigella dysenteriae* positions |  |
|  | SG1F | CGAGCTGATCTGCGCAGCGA | 921-940 | SG1 |
|  | SG1R | CAGACGGTCGTTAGTTGGGT | 1080-1061 |  |
|  | SG2F | CGAGCCGCCGACTCGTGAAGCA | 1118-1140 | SG2 |
|  | SG2R | GCTTTTTCATAACATCAATGATGT | 1294-1271 |  |
| rpoB bacterial gene for S*treptococcus pneumoniae* |  |  | *Streptococcus pneumoniae* positions |  |
|  | STR | GTTAGCCTGAGCTACAGTATATTCA | 1790-1761 | STshort |
|  | STF  (short) | GTCATCTTACGGACACTTGAACAA | 1644-1785 |  |
|  | STF  (long) | TAACTTGTCATCTTACGGACACTTGAACAA | 1638-1785 | STlong |
