# Supplementary material for: Molecular Identification of Bacteria by Total Sequence Screening: Determining the Cause of Death in Ancient Human Subjects
Source: PLoS One. 2011 Jul 13;6(7):e21733. doi: 10.1371/journal.pone.0021733 (PMC3135582; doi:10.1371/journal.pone.0021733)
Supplement: Text S1 — Ancient DNA analysis informations (DOC) [file pone.0021733.s007.doc]

Molecular identification of bacteria by total sequence screening:
determining the cause of death in ancient human subjects.

Catherine Thèves^1,2*^, Alice Senescau^2^ , Stefano Vanin^4^, Christine Keyser^1^, François Xavier Ricaut^1^, Anatoly N. Alekseev^5^, Henri Dabernat^1,6^, Bertrand Ludes^1,3^, Richard Fabre^2^ , Eric Crubézy^1^.

* Laboratoire AMIS, UMR5288, Université Toulouse IIII/ CNRS/Université de Strasbourg, Toulouse, France.

email: ctheves@cict.fr

**Contact between autochthonous Siberian and European populations**

The Russian arrival into Yakutia in 1632, starting with the construction of the Russian fort in Yakutsk, had an important impact on Yakut populations, since it initiated a long process of acculturation. Siberian populations exclusively wore fur and leather, as they offered an effective protection against the cold climate. When the Russians arrived during the 17^th^ century, they exploited Siberians by introducing a tax, which autochthonous populations had to pay in fur. The mass conversion to Christianity, from 1760 until 1820, saw the proliferation of burials in Yakutia, which had previously been rare. The burials that have been discovered, dating from the first part of this period (15^th^ century to 1689), are typically those of the elite Siberian class. It can be envisaged that the Europeans (Russian soldiers, Cossacks and merchants) brought new diseases that were rapidly transmitted to autochthonous populations with no immunological protection. To explore this hypothesis, it is necessary to study additional graves of the Siberian elite class from this period (the only subjects buried at this time), in order to measure the impact of the potential epidemics on the Yakut populations [S1,S2].

**Decontamination of the laboratory under positive pressure dedicated to ancient DNA**

The work related to the amplification of ancient DNA products was conducted in a laboratory dedicated to ancient DNA (lab.3) under positive pressure (Cleanrooms, UK; <http://www.connect2cleanrooms.com/index.html>), where no modern bacterial strains, or primers and PCR products used in protocols on modern strains had been introduced. Work surfaces, two laminar flow hoods (hood 1 dedicated to preparations without DNA; hood 2 dedicated to ancient DNA samples) and small equipments (e.g. pipettes, bench centrifuges, vortex) were all decontaminated in three phases before use: cleaning with bleach, 80% ethanol and finally with a fungicide/bactericide (ANIOS D.D.S). Between each cleaning phase the laboratory and hoods were exposed to 45 minutes of UV light. The small equipments, including tips and PCR tubes, were sterilized. Before each use, work surfaces and small equipments were cleaned for a second time with a fungicide/bactericide (ANIOS D.D.S) and with DNAaway (Dustcher). The interiors and surfaces of the hoods, the interior of the tent, and small equipments were exposed to UV light for 45 minutes before and after all the PCR mix preparations. During the study, access to the laboratory was restricted to one person who worked in full protective clothing, a mask, glasses and gloves.

**Decontamination of PCR products for the *16S rDNA* gene amplification**

In lab.2, the first step in the decontamination of the PCR reaction mix was performed according to the protocol established by Tran-Hung et al. (2007) [S3]. Manipulations were performed under an isolated laminar flow hood (Biocap DNA/RNA, Captair by Erlab, Fisher), independent of other laboratory activities. We aligned the sequences of the *16s rDNA* gene of the different bacterial species that could be potential laboratory contaminants, including *Pseudomonas* sp., *Xanthomonas* sp., *Ralstonia* sp. and *Bacillus* sp. [S3]. Particular attention was given to *Eschericia* sp. because its DNA is present at insignificant levels in the Taq LD polymerase; the supplier (Applied Biosystems) guarentees less than ten copies of the bacterial *16S rDNA* gene in 5U. However, this number of copies is sufficient to create false positives in the PCR blanks with 40 cycles and thus, needs to be eliminated before amplifications of the ancient bacterial DNA [S4]. The restriction enzyme maps established (on the site NEBcutter: http://tools.neb.com/NEBcutter2/) showed that the HaeIII and AclI (Biolabs) enzyme sites were present in several locations on the target segments. We therefore used these two enzymes in conjunction with Buffer 4 (Biolabs) in the PCR mix containing all the necessary reagents, except the DNA samples, which were added after the decontamination step.

After determining the optimal conditions of this protocol in lab.2, the protocol was conducted in a laboratory dedicated to ancient DNA (lab.3), with all the dedicated reagents and equipment for ancient DNA analysis.

**Contamination controls**

A blank PCR was run for every three PCR tubes for each sample [S3] and the extraction blanks were examined systematically. Apart from the assigned ancient DNA primers used under hood 1 for the preparation of the PCR mix, no DNA samples were introduced. After adding the mix to each PCR tube under hood 1, the ancient DNA samples were added under hood 2. No PCR tubes were opened at the same time during these two steps. Only one researcher worked on these samples in lab.3. No positive control was included into any of the pre-PCR preparation steps or to the PCR amplifications of the ancient DNA samples in the ancient DNA laboratory.

**The use of positive controls for *rpoB* gene analysis for the three pathogens**

Positive controls were only added to the relevant PCR tubes in a room dedicated to modern DNA in lab.3, physically separated from the ancient DNA PCR tubes. The positive controls were put in different thermocycler allocated to modern DNA in a different PCR room to the ancient DNA. Positive control was only used to evaluate the correct amplification of the PCR product derived from the common PCR mix and as a comparative band in the agarose gel. Again, the cloning of the positive control PCR products for the segments bor1 and bor2 showed that the sequences were identical to the *Bordetella pertussis* strain Tohama I (NC_002929.2). For further explanation of the PCR results, with regard to the use of positive controls for research on pathogens, see Text S3.

**DNA extracts and multiple independent PCRs**

Two independent extractions for each tooth and lung tissue sample were performed when samples were available. Both lung tissue samples (A and B) were tested in two independent PCR amplifications (1 and 2 for each A and B) when amplification of the specific pathogen (*Bordetella pertussis*) was positive in lung tissues from boul 1.

Amplified bor1 segments for *B. Pertussi*s were directly sequenced for the four amplifications (A1, A2; B1, B2; Text S4) and cloned and sequenced from the first amplification (A1).

**Cloning and sequencing**

The post-PCR laboratory is situated in a different building from the laboratory dedicated to ancient DNA (lab.3). Protocol post-PCR steps were performed in a post-PCR room dedicated to cloning and sequencing. When the PCR product bands were visible in the agarose gels, they were extracted with MinElute Kit (Qiagen) and sequenced directly. 15µl of the remaining PCR products were purified and cloned in pGEM®-T Easy Vector Systems II according to the manufacturer’s instructions (Promega).

Vector DNA purifications for each clone were realised with QIAprep Spin Miniprep kit (Qiagen), according to the manufacturer’s instructions. For each amplification product we attempted to obtain at least ten clones, in order to gain maximum sequence information.

Cloned products were amplified with SP6 and T7 primers to obtain both strands of the PCR cloned products (pGEM®-T Easy Vector, Promega) and sequencing was carried out with Big-Dye Terminator V3.1 Cycle sequencing kit (Applied Biosystems) according to the manufacturer’s specifications. The sequence reaction products were purified using Sephadex G-50 Fine (Sigma-Aldrich) and analysed by capillary electrophoresis on ABI Prism 3170 Genetic Analyser (Applied Biosystems) in the genomic technical platform PlaGe (Genopole, Toulouse, France).

S1. Crubézy E, AN. A (2007) Chamane Kyss, jeune fille des glaces. Paris. 167 p.

S2. Crubezy E, Amory S, Keyser C, Bouakaze C, Bodner M, et al. (2010) Human evolution in Siberia: from frozen bodies to ancient DNA. BMC Evol Biol 10: 25.

S3. Tran-Hung L, Tran-Thi N, Aboudharam G, Raoult D, Drancourt M (2007) A new method to extract dental pulp DNA: application to universal detection of bacteria. PLoS One 2: e1062.

S4. Corless CE, Guiver M, Borrow R, Edwards-Jones V, Kaczmarski EB, et al. (2000) Contamination and sensitivity issues with a Real-Time Universal 16S rRna PCR. Journal of clinical Microbiology 38: 1474-1752.
