# Supplementary material for: Molecular Identification of Bacteria by Total Sequence Screening: Determining the Cause of Death in Ancient Human Subjects
Source: PLoS One. 2011 Jul 13;6(7):e21733. doi: 10.1371/journal.pone.0021733 (PMC3135582; doi:10.1371/journal.pone.0021733)
Supplement: Text S2 — Alignments of the 16S rDNA sequences from boul 1, OYA and OYB subjects and the matched sequences recorded in the Genbank database. (DOC) [file pone.0021733.s008.doc]

**Molecular identification of bacteria by total sequence screening: determining the cause of death in ancient human subjects.**

Catherine Thèves^1,2*^, Alice Senescau^2^ , Stefano Vanin^3^, Christine Keyser^1^, François Xavier Ricaut^1^, Anatoly N. Alekseev^5^, Henri Dabernat^1,6^, Bertrand Ludes^1,4^, Richard Fabre^2^ , Eric Crubézy^1^.

* Laboratoire AMIS, UMR5288, Université Toulouse IIII/ CNRS/Université de Strasbourg, Toulouse, France.

email: [ctheves@cict.fr](mailto:ctheves@cict.fr)

S2a: *16S rDNA* sequences from boul 1 (bo1), from teeth extracts (n2) from M1 segment (M1), for 3 clones (c2, c8 and c12).

10 20 30 40 50 60 70

....|....| ....|....| ....|....| ....|....| ....|....| ....|....| ....|....|

bo1n2M1c2 GCTCGTGTCG TGAGATGTTG GGTTAAGTCC CGCAACGAGC GCAACCCTTG TCATTAGTTG CTACGAAAGG

bo1n2M1c8 .......... .......... .......... .......... .......... .......... ..........

bo1n2M1c12 .......... .......... .......... .......... .......... .......... ..........

NZ_GG770424.1 Achromobacter piechaudii .......... .......... .......... .......... .......... .......... ..........

NC_014640.1 Achromobacter xyloxydans .......... .......... .......... .......... .......... .......... ..........

NC_010170.1 Bordetella petrii .......... .......... .......... .......... .......... .......... ..........

NC_010645.1 Bordetella avium .......... .......... .......... .......... .......... .......... ..........

NC_002927.3 Bordetella bronchiseptica .......... .......... .......... .......... .......... .......... ..........

NC_002928.3 Bordetella parapertussis .......... .......... .......... .......... .......... .......... ..........

NC_002929.2 Bordetella pertussis .......... .......... .......... .......... .......... .......... ..........

80 90 100

....|....| ....|....| ....|....| .

bo1n2M1c2 GCACTCTAAT GAGACTGC-G GTGACAAACC G 100

bo1n2M1c8 .......... ........C. .......... . 101

bo1n2M1c12 .......... ........C. .......... . 101

NZ_GG770424.1 Achromobacter piechaudii .......... ........C. .......... . 101

NC_014640.1 Achromobacter xyloxydans .......... ........C. .......... . 101

NC_010170.1 Bordetella petrii .......... ........C. .......... . 101

NC_010645.1 Bordetella avium .......... ........C. .......... . 101

NC_002927.3 Bordetella bronchiseptica .......... ........C. .......... . 101

NC_002928.3 Bordetella parapertussis .......... ........C. .......... . 101

NC_002929.2 Bordetella pertussis .......... ........C. .......... . 101

S2b: *16S rDNA* sequences from boul 1 (bo1) subject, from teeth extracts (n2) from P2 segment (P2), for 2 clones (c6 and c13).

10 20 30 40 50 60 70

....|....| ....|....| ....|....| ....|....| ....|....| ....|....| ....|....|

bo1P2c6 CAAGTCCTCA TGGCCCTTAT GGGTAGGGCT TCACACGTCA TACAATGGTC GGGACAGAGG GTCGCCAACC

bo1P2c13 .......... .......... .......... .......... .......... .......... ..........

NZ_GG770424.1 Achromobacter piechaudii .......... .......... .......... .......... .......... .......... ..........

NC_014640.1 Achromobacter xyloxydans .......... .......... .......... .......... .......... .......... ..........

NC_010645.1 Bordetella avium .......... .......... .......... .......... .......... .......... ..........

NC_002927.3 Bordetella bronchiseptica .......... .......... .......... .......... .......... .......... ..........

NC_002928.3 Bordetella parapertussis .......... .......... .......... .......... .......... .......... ..........

NC_010170.1 Bordetella petri .......... .......... .......... .......... .......... .......... .CT.......

NC_002929.2 Bordetella pertussis .......... .......... .......... .......... .......... .......... ..T.......

80 90 100 110 120 130 140

....|....| ....|....| ....|....| ....|....| ....|....| ....|....| ....|....|

bo1P2c6 CGCAAGGGGG AGCCAATCCC AGAAACCCGA TCGTAGTCCG GATTGCAGGC TGCAACTCGC CTGCATGAAG

bo1P2c13 .......... ...T...... .T...G..AG .......... ...C...... .......... ..........

NZ_GG770424.1 Achromobacter piechaudii ...G...... .......... .......... .......... ...C....T. .........A ....G.....

NC_014640.1 Achromobacter xyloxydans ...G...... .......... .......... .......... ...C....T. .........A ....G.....

NC_010645.1 Bordetella avium ...G...... .......... .......... .......... ...C....T. .........A ....G.....

NC_002927.3 Bordetella bronchiseptica ...G...... .......... .......... .......... ...C....T. .........A ....G.....

NC_002928.3 Bordetella parapertussis ...G...... .......... .......... .......... ...C....T. .........A ....G.....

NC_010170.1 Bordetella petri .......... .......... .......... .......... ...C....T. .........A ....G.....

NC_002929.2 Bordetella pertussis ...G...... .......... .........G .......... ...C....T. .........A ....G.....

150 160 170

....|....| ....|....| ....|....| ....|.

bo1P2c6 TCGGAATCGC TAGTAATCGC GGATCAGAAT GCCGCG 176

bo1P2c13 .......... .......... .......... ...... 176

NZ_GG770424.1 Achromobacter piechaudii .......... .......... .......C.. .T.... 176

NC_014640.1 Achromobacter xyloxydans .......... .......... .......C.. .T.... 176

NC_010645.1 Bordetella avium .......... .......... .......C.. .T.... 176

NC_002927.3 Bordetella bronchiseptica .......... .......... .......C.. .T.... 176

NC_002928.3 Bordetella parapertussis .......... .......... .......C.. .T.... 176

NC_010170.1 Bordetella petri .......... .......... .......C.. .T.... 176

NC_002929.2 Bordetella pertussis .......... .......... .......C.. .T.... 176

S2c: *16S rDNA* sequences from OYA and OYB subjects, from teeth extracts, from M2 segment (M2), for 3 clones (c1, c2 and c11).

10 20 30 40 50 60 70

....|....| ....|....| ....|....| ....|....| ....|....| ....|....| ....|....|

OYAM2c1 CCATGAAGTC GGAATCGCTA GTAATCGTGG ATCAGAATGT CACGGTGAAT ACGTTCCCGG GTCTTGTACA

OYAM2c2 .......... .......... .......... .......... .......... .......... ..........

OYBM2c11 .......... .......... .......... .......... .......... .......... ..........

NC_007606.1 Shigella dysenteriae .......... .......... .......... .......... .......... .......... .C........

80 90

....|....| ....|....| ....

OYAM2c1 CACCGCCCGT CACAC-ATGG GAGT 93

OYAM2c2 .......... .....-.... .... 93

OYBM2c11 .......... .....-.... .... 93

NC_007606.1 Shigella dysenteriae .......... .....C.... .... 93

S2d: *16S rDNA* sequences from OYB subject, from teeth extracts, from P8 segment (P8), for 1 clones (c6).

10 20 30 40 50 60 70

....|....| ....|....| ....|....| ....|....| ....|....| ....|....| ....|....|

OYBP8c6 TGCGGGACTT AACCCAACAT CTCACGACAC GAGCTGACGA CAACCATGCA CCACCTGTGT CCTCTGTACC

NC_011072.1 Streptococcus pneu .......... .......... .......... .......... .......... ........CA .......-..

NC_003028.3 Streptococcus pneu .......... .......... .......... .......... .......... ........CA .......-..

NC_010582.1 Streptococcus pneu .......... .......... .......... .......... .......... ........CA .......-..

NC_010380.1 Streptococcus pneu .......... .......... .......... .......... .......... ........CA .......-..

80 90 100 110 120 130 140

....|....| ....|....| ....|....| ....|....| ....|....| ....|....| ....|....|

OYBP8c6 CGAAGGTAAA GATCTATCTC TAGACCGGTC AGAGGCATGT CAAGCCCTGG TAAGGTTCTT CGCGTTGCTT

NC_011072.1 Streptococcus pneu ......A... C-........ ....G..... .....G.... ....A..... .......... ..........

NC_003028.3 Streptococcus pneu ......-... .C........ ....G..... .....G.... ....A..... .......... ..........

NC_010582.1 Streptococcus pneu ......-... .C........ ....G..... .....G.... ....A..... .......... ..........

NC_010380.1 Streptococcus pneu ......-... .C........ ....G..... .....G.... ....A..... .......... ..........

150 160

....|....| ....|....| .

OYBP8c6 CGAATTAAAC CACATGCTCC A 161

NC_011072.1|Streptococcus pneu .......... .......... . 159

NC_003028.3 Streptococcus pneu .......... .......... . 159

NC_010582.1|Streptococcus pneu .......... .......... . 159

NC_010380.1|Streptococcus pneu .......... .......... . 159
