# Supplementary material for: Molecular Identification of Bacteria by Total Sequence Screening: Determining the Cause of Death in Ancient Human Subjects
Source: PLoS One. 2011 Jul 13;6(7):e21733. doi: 10.1371/journal.pone.0021733 (PMC3135582; doi:10.1371/journal.pone.0021733)
Supplement: Text S3 — Alignments of the rpoB sequences from boul 1 subject with the sequences of B. pertussis, B. bronchiseptica, B. parapertussis, and B. petrii recorded in the Genbank database. (DOC) [file pone.0021733.s009.doc]

Molecular identification of bacteria by total sequence screening:
determining the cause of death in ancient human subjects.

Catherine Thèves^1,2*^, Alice Senescau^2^ , Stefano Vanin^3^, Christine Keyser^1^, François Xavier Ricaut^1^, Anatoly N. Alekseev^5^, Henri Dabernat^1,6^, Bertrand Ludes^1,4^, Richard Fabre^2^ , Eric Crubézy^1^.

* Laboratoire AMIS, UMR5288, Université Toulouse IIII/ CNRS/Université de Strasbourg, Toulouse, France.

email: ctheves@cict.fr

**Other pathogens: *Shigella dysenteriae* and *Streptococcus pneumoniae***

The ancient DNA extracts from the other subjects showed no amplifications of the *rpoB* gene sequences. The M2 segment identified from *16S rDNA* sequence as *Shigella dysenteriae* was represented by three clones from two subjects, OYA and OYB (Table S1b and c; Text S2c). These three clones were detected from a smaller M2 segment and thus, could be less reliable since it is located in conserved region C7 of the *16S* *rDNA* gene [S1]. This region presents a high degree of identity among bacterial species and potentially does not show informative bacterial sequences. *Streptococcus pneumoniae* was only represented by one *16S rDNA* clone from OYB (Table S2c; Text S2d) and one sequence from OYA with a degree of identity of 92% (data not shown). The three subjects from the multiple grave (Oyogosse Tumula; Fig.S3) were found in skeletal states and therefore other criteria, for example tissue or histological sections to reinforce the *16S rDNA* results, were absent. The identification of both pathogens should be interpreted with caution, due to the lack of results from the *rpoB* gene.

**Segment bor2 from *rpoB* gene for *Bordetella sp.***

Relating to the amplified bor2 segment from the teeth and lung tissues of boul 1, the degree of identity with *B. pertussis* was lower (93-94%) and had numerous mutations (transversions and transitions). Indeed, the typical profile C →T/ G →A [S2,S3] was not the only profile observed; tranversions were present in all of the clones from teeth or lung tissue samples. The patterns of base changes (transitions and tranversions) and the number of mutations (12 on average) involving different positions between clones of the same sample led to the conclusion that this was an amplification of a chimeric sequence (data not shown). It is known that chimeric sequences are created during the PCR process through the Taq polymerase (despite its high fidelity) or due to the lower specificity of the designed primers [S2-S5] in ancient DNA. In addition, the calculation of damage rates during post-mortem degradation resulted in very high values [S2,S6].

To complete the analysis of variation for the bor2 sequence, we searched for conserved domains of the *rpoB* gene in the *B. pertussis* strain (<http://www.ncbi.nlm.nih.gov/Structure/cdd/wrpsb.cgi?seqinput=NP_878932.1>; [S7,S9]). The bor2 segment is situated within a conserved region of the *rpoB* gene (cd 00653 [S7]) and therefore should show little variation.

**Differential conservation of human and bacterial DNA in specific tissues**

Concerning the differential conservation of human and bacterial DNA in the various tissues, amplifications with Quantifiler® Human DNA Quantification Kit and AmpFlSTR® Profiler Plus™ Kit (Applied Biosystems) gave human nuclear DNA quantity and human autosomal STR profiles for teeth samples, but not for lung tissue samples. In the search for pathogenic bacterial DNA, the *16S rDNA* gene was amplified in the teeth samples and the *rpoB* gene in the lung tissue samples from the boul 1 subject. This highlights the differential conservation of human and bacterial DNA in tissues, which is contrary to what might be expected. Taylor et al. [S10] proposed that in older samples human cellular DNA is less likely to persist compared to bacterial DNA.

The M1 and P2 segments of the *16S rDNA* gene were amplified for the teeth samples from boul 1 with five clones with high degrees of identity to *Bordetella sp.* (≥ 95%; Table S2a). It should be noted that the bor1 segment was only amplified for the lung tissue sample of the subject boul 1. In fact, after several analyses of the boul 1 teeth samples, we could not perform amplification of the *rpoB* gene due to a lack of DNA extract. For the lung tissue of boul 1, the amplification of the gene *rpoB* was performed on two independent extractions and the reproducibility of the PCR was made two times for each extraction (sequences are presented in Text S4). The *16S rDNA* gene is often present in multiple copies in bacteria, and perhaps is easier to detect in teeth. Moreover, the probability that more *Bordetella* sequences were present in lung tissue than teeth samples is higher for a diseased subject. In comparison with the lung tissue of the boul 2 subject, this sample never amplified for the bor1 or bor2 segments of the *rpoB* gene from *Bordetella sp*.

**The use of positive controls in the identification of *Bordetella pertussis, Shigella dysenteriae* and *Streptococcus pneumoniae***

Following Drancourt and Raoult [S11], positive controls were never present in the ancient DNA laboratory and their purpose is explained in Text S1. Maximum precautions were taken against potential contamination: multiple blanks during PCR cycling and the separation of ancient samples and modern controls in different thermocyclers at different times. For the amplification of bor1 segment, the positive control used for *B. pertussis* presented a sequence that was 100% identical to *B. pertussis* Tohama I (NC_002929.2), with a T at position 2973, demonstrating that cross-contamination had not occurred between the ancient sample and the positive control. Similarly, the bor2 segment of the positive control was 100% identical to the *B. pertussis* strain (NC_002929.2), whereas the bor2 amplified segment from the ancient sample presented chimeric sequences (as discussed above). As stated by Taylor et al. [S10], if the aim is to quantify or amplify ancient DNA, it is possible to use a modern standard comparison, even if it is necessary that it is done after the other amplifications. In our study, during the PCR preparations, in addition to the target ancient sample (boul 1, teeth and lung tissue) we tested a lung tissue sample from boul 2, a PCR blank for every three PCR tubes, extraction blanks and a tube for the positive control. Positive control DNA was added last and amplified independently in a separate laboratory, and was never in direct contact with PCR tubes containing ancient DNA (see S1 text; [S10]). Similarly, positive controls were used for *S. pneumoniae* and *S. dysenteriae*; amplifications were never positive in the ancient samples or in the multiple blanks (extraction and PCR blanks). Positive controls were only added to agarose gels and allowed us to have a standard control during electrophoresis migration.

S1. Petrosino JF, Highlander S, Luna RA, Gibbs RA, Versalovic J (2009) Metagenomic pyrosequencing and microbial identification. Clin Chem 55: 856-866.

S2. Hofreiter M, Jaenicke V, Serre D, von Haeseler A, Paabo S (2001) DNA sequences from multiple amplifications reveal artifacts induced by cytosine deamination in ancient DNA. Nucleic Acids Res 29: 4793-4799.

S3. Gilbert MT, Willerslev E, Hansen AJ, Barnes I, Rudbeck L, et al. (2003a) Distribution patterns of postmortem damage in human mitochondrial DNA. Am J Hum Genet 72: 32-47.

S4. Cooper A, Lalueza-Fox C, Anderson S, Rambaut A, Austin J, et al. (2001) Complete mitochondrial genome sequences of two extinct moas clarify ratite evolution. Nature 409: 704-707.

S5. Gilbert MT, Hansen AJ, Willerslev E, Rudbeck L, Barnes I, et al. (2003b) Characterization of genetic miscoding lesions caused by postmortem damage. Am J Hum Genet 72: 48-61.

S6. Gilbert MT, Binladen J, Miller W, Wiuf C, Willerslev E, et al. (2007) Recharacterization of ancient DNA miscoding lesions: insights in the era of sequencing-by-synthesis. . Nucleic Acids Research 35: 1-10.

S7. Parkhill J, Sebaihia M, Preston A, Murphy LD, Thomson N, et al. (2003) Comparative analysis of the genome sequences of Bordetella pertussis, Bordetella parapertussis and Bordetella bronchiseptica. Nat Genet 35: 32-40.

S8. Marchler-Bauer A, Anderson JB, Chitsaz F, Derbyshire MK, DeWeese-Scott C, et al. (2009) CDD: specific functional annotation with the Conserved Domain Database. Nucleic Acids Res 37: D205-210.

S9. Marchler-Bauer A, Bryant SH (2004) CD-Search: protein domain annotations on the fly. Nucleic Acids Res 32: W327-331.

S10. Taylor GM, Mays SA, Hugget JF (2010) Ancient DNA (aDNA) studies of man and microbes: general similarities, specific differences. International Journal of Osteoarchaeology 20: 747-751.

S11. Drancourt M, Raoult D (2005) Palaeomicrobiology: Current issues and perspectives. Nature Reviews Microbiology 3: 23-35.
