# Supplementary material for: Molecular Identification of Bacteria by Total Sequence Screening: Determining the Cause of Death in Ancient Human Subjects
Source: PLoS One. 2011 Jul 13;6(7):e21733. doi: 10.1371/journal.pone.0021733 (PMC3135582; doi:10.1371/journal.pone.0021733)
Supplement: Text S4 — Supplementary data of the obtained results from B. pertussis, S. dysenteriae and S. pneumoniae. (DOC) [file pone.0021733.s010.doc]

**Molecular identification of bacteria by total sequence screening: determining the cause of death in ancient human subjects.**

Catherine Thèves^1,2*^, Alice Senescau^2^ , Stefano Vanin^3^, Christine Keyser^1^, François Xavier Ricaut^1^, Anatoly N. Alekseev^5^, Henri Dabernat^1,6^, Bertrand Ludes^1,4^, Richard Fabre^2^ , Eric Crubézy^1^.

*Laboratoire AMIS, UMR5288, Université Toulouse IIII/ CNRS/Université de Strasbourg, Toulouse, France. email: [ctheves@cict.fr](mailto:ctheves@cict.fr)

*rpoB* sequences from boul 1 (bo1), from two lung tissue extracts (n8: A or B) from bor1 segment (x1), for 10 clones (c1 to c12 from PCR A1 amplification) and with two independent PCR (A1, A2, B1, B2).

*B.pertussis* *rpoB* gene position 1 90 900 910 920 930 940 950

....|....| ....|....| ....|....| ....|....| ....|....| ....|....| ....|....|

NC_002929.2|B.pertussis 2881 CGTTATCGCC AGGACCTCAA CGACCAGCTG CGCATCGTCG AGAACGACCA GTTCGACCGT ATCGAGAAGA

bo1n8A1x1c1 .......... .......... .......... .......... .......... .......... ..........

bo1n8A1x1c2 .......... .......... .......... .......... .......... .......... ..........

bo1n8A1x1c3 .......... .......... .......... .......... .......... .......... ..........

bo1n8A1x1c4 .......... .......... .......... .......... .......... .......... ..........

bo1n8A11c7 .......... .......... .......... .......... .......... .......... ..........

bo1n8A1x1c8 .......... .......... .......... .......... .......... .......... ..........

bo1n8A1x1c9 .......... .......... .......... .......... .......... .......... ..........

bo1n8A1x1c12 .......... .......... .......... .......... .......... .......... ..........

bo1n8A1x1c6 .......... .......... .......... .......... .......... .......... ..........

bo1n8A1x1c5 .......... .......... .......... .......... .......... .......... ..........

bo1n8A1x1 ---....... .......... .......... .......... .......... .......... ..........

bo1n8A2x1 ---------- ---------. .......... .......... .......... .......... ..........

bo1n8B1x1 -----..... .......... .......... .......... .......... .......... ..........

bo1n8B2x1 -......... .......... .......... .......... .......... .......... ..........

NC_002927.3|B.bronchiseptica 2906 .......... .......... .......... .......... .A........ .......... ..........

NC_002928.3|B.parapertussis 2906 .......... .......... .......... .......... .A........ .......... ..........

NC_010170.1|B.petrii 2885 ---....... .A.....G.. .......... .......... .......... .......... .........C

960 970 980 990 3000 10

....|....| ....|....| ....|....| ....|....| ....|....| ....|.-... |....|....

NC_002929.2|B.pertussis TGCTGGTCGG CAAGACCGTC AATGGCGGCC CGCGCAAGCT GGCCAAGGGC GCCACC-TTG ACCAAGGCCT

bo1n8A1x1c1 .......... .......... ..C....... .......... .......... ......-... ..........

bo1n8A1x1c2 .......... .......... ..C....... .......... .......... ......-... ..........

bo1n8A1x1c3 .......... .......... ..C....... .......... .......... ......-... ..........

bo1n8A1x1c4 .......... .......... ..C....... .......... .......... ......-... ..........

bo1n8A1x1c7 .......... .......... ..C....... .......... .......... ......-... ..........

bo1n8A1x1c8 .......... .......... ..C....... .......... .......... ......-... ..........

bo1n8A1x1c9 .......... .......... ..C....... .......... .......... ......-... ..........

bo1n8A1x1c12 .......... .......... ..C....... .......... .......... ......-... ..........

bo1n8A1x1c6 .......... .......... ..C....... .......... .......... ......-... ..........

bo1n8A1x1c5 .......... ....G..... ..C....... .......... .......... ......-... ..........

bo1n8A1x1 .......... .......... ..C....... .......... .......... ......-..- ----------

bo1n8A2x1 .......... .......... ..C....... .......... .......... ......-... ..........

bo1n8B1x1 .......... .......... ..C....... .......... .......... ......-... ..........

bo1n8B2x1 .......... .......... ..C....... .....----- ---------- ---------- ----------

NC_002927.3|B.bronchiseptica .......... ....G..... .......... .......... .......... ......-... ..........

NC_002928.3|B.parapertussis .......... ....G..... .......... .......... .......... ......-... ..........

NC_010170.1|B.petrii .....A.... .......... ..C....... .......... .......... ......A..- ........G.

20

|..-..|.

ref|NC_002929.2|B.pertussis ACC-TGGC 3026

bo1n8A1x1c1 ...-.... 146

bo1n8A1x1c2 ...-.... 146

bo1n8A1x1c3 ...-.... 146

bo1n8A1x1c4 ...-.... 146

bo1n8A1x1c7 ...-.... 146

bo1n8A1x1c8 ...-.... 146

bo1n8A1x1c9 ...-.... 146

bo1n8A1x1c12 ...-.... 146

bo1n8A1x1c6 ...C.... 147

bo1n8A1x1c5 ...-.... 146

bo1n8A1x1 -------- 125

bo1n8A2x1 ...-.... 127

bo1n8B1x1 ...-.... 141

bo1n8B2x1 -------- 104

NC_002927.3|B.bronchiseptica ...-.... 3051

NC_002928.3|B.parapertussis ...-.... 3051

NC_010170.1|B.petrii ...-.... 3027
